# Diversity and Bioprospection of Gram-positive Bacteria Derived from a Mayan Sinkhole

**DOI:** 10.1007/s00248-024-02392-1

**Published:** 2024-05-29

**Authors:** Julian L. Wissner, José Carlos Parada-Fabián, Norma Angélica Márquez-Velázquez, Wendy Escobedo-Hinojosa, Susana P. Gaudêncio, Alejandra Prieto-Davó

**Affiliations:** 1 Unidad de Química en Sisal, Facultad de Química, Universidad Nacional Autónoma de México, Puerto de abrigo s/n, Sisal, Yucatán, 97356 México; 2 Associate Laboratory i4HB, Institute for Health and Bioeconomy, NOVA Faculty of Sciences and Technology, NOVA University of Lisbon, Lisbon, 2819-516 Portugal; 3 Applied Molecular Biosciences Unit, Chemistry and Life Sciences Departments, NOVA Faculty of Sciences and Technology, UCIBIO, NOVA University of Lisbon, Lisbon, 2819-516 Portugal

**Keywords:** Gram-positive Bacteria, Agar Plate Assays, Hydrolytic Enzymes, 16S rRNA, Antimicrobial Activity

## Abstract

Water-filled sinkholes known locally as cenotes, found on the Yucatán Peninsula, have remarkable biodiversity. The primary objective of this study was to explore the biotechnological potential of Gram-positive cultivable bacteria obtained from sediment samples collected at the coastal cenote Pol-Ac in Yucatán, Mexico. Specifically, the investigation aimed to assess production of hydrolytic enzymes and antimicrobial compounds. 16 S rRNA gene sequencing led to the identification of 49 Gram-positive bacterial isolates belonging to the phyla Bacillota (*n* = 29) and Actinomycetota (*n* = 20) divided into the common genera *Bacillus* and *Streptomyces*, as well as the genera *Virgibacillus, Halobacillus, Metabacillus, Solibacillus, Neobacillus, Rossellomorea, Nocardiopsis* and *Corynebacterium*. With growth at 55ºC, 21 of the 49 strains were classified as moderately thermotolerant. All strains were classified as halotolerant and 24 were dependent on marine water for growth. Screening for six extracellular hydrolytic enzymes revealed gelatinase, amylase, lipase, cellulase, protease and chitinase activities in 93.9%, 67.3%, 63.3%, 59.2%, 59.2% and 38.8%, of isolated strains, respectively. The genes for polyketide synthases type I, were detected in 24 of the strains. Of 18 strains that achieved > 25% inhibition of growth in the bacterial pathogen *Staphylococcus aureus* ATCC 6538, 4 also inhibited growth in *Escherichia coli* ATCC 35,218. Isolates *Streptomyces* sp. NCA_378 and *Bacillus* sp. NCA_374 demonstrated 50–75% growth inhibition against at least one of the two pathogens tested, along with significant enzymatic activity across all six extracellular enzymes. This is the first comprehensive report on the biotechnological potential of Gram-positive bacteria isolated from sediments in the cenotes of the Yucatán Peninsula.

**Supplementary Information:**

The online version contains supplementary material available at 10.1007/s00248-024-02392-1.

## Introduction

The Yucatán Peninsula in the Gulf of Mexico is a karst landscape with many natural flooded sinkholes, formed by the collapse of limestone bedrock and known locally as cenotes [1]. These cenotes are of profound cultural significance as they served as vital water sources for the ancient Maya civilization and were revered as sacred sites for their ritual activities [2]. Their unexploited biotechnological potential now requires urgent investigation [3], for example as source of novel halotolerant bacteria possessing antimicrobial properties [4].

Cenotes close to the coast show a saltwater intrusion beneath the freshwater surface, and this creates a marked shallow halocline [5]. Low concentrations of oxygen, high concentrations of sulfate and other nutrients, and stable water temperature, make them ideal habitats for the growth of distinctive and diverse species of bacteria [5, 6]. Their bacterial communities not only help in the decomposition of organic matter, but are also crucial in biogeochemical cycling, nutrient recycling, and the delicate ecosystem equilibrium [7, 8]. Therefore, they may produce a range of enzymes with biotechnological potential, such as extracellular carbohydrases, lipases and proteases [9, 10].

Since extracellular enzymes can break down complex molecules into simpler ones, they are of high commercial value in industrial biotechnology [11, 12], with applications such as food and beverage production, biofuel generation, and waste treatment. Carbohydrases, such as amylases, cellulases and chitinases, hold a significant share in the global industrial enzymes market, which was worth 6.0 billion US$ in 2021 [13]. For example, amylases are used to hydrolyze starch into polymers composed of glucose units, which can then be fermented to produce bioethanol [14]. Cellulases can break down plant biomass such as cellulose into simple sugars, which can be further processed into biofuels [15] Lipases are used in the production of detergents, cosmetics, and pharmaceuticals [16], and proteases are used for protein hydrolysis in food technology [17]. Chitinases have potential particularly in the agro-industry, but also in wastewater treatment, the food industry, cosmetics, and medicine [18]. The unexplored microbial biodiversity found in cenotes offers the potential discovery of novel enzymes with applications in these industries. Bacteria from coastal cenote sediments are influenced by marine-like conditions; they produce halotolerant extracellular enzymes, which are in high demand in the biotechnological industry [19].

Another application of bacteria from this ecological niche is the production of antibiotics. Bacteria from cenotes produce antimicrobial compounds with potential medical applications [3, 4]. Some members of the phyla Actinomycetota and Bacillota have emerged as prominent sources of antimicrobial compounds [20] accounting for > 50% of all antimicrobial activities reported in recent decades, and they merit further exploration in unusual habitats such as cenotes. Actinomycetota produce many substances relevant to biotechnology, agriculture and medicine, including the majority of currently employed antibiotics [21]. Indeed, > 75 antibiotics isolated from Actinomycetota are used in human therapy [22]. Due to the increasing resistance of clinically important microorganisms to therapeutic drugs [23], and the steady decline in the discovery of new antibiotics [24], the need for novel antibiotics rises steadily. Polyketides such as the glycopeptide vancomycin, the lipopeptide daptomycin and the macrolide erythromycin have important antibiotic effects [25], notably against Gram-positive bacteria, and can also have antitumor and immunosuppressive properties [26]. The biosynthesis of polyketides is catalyzed by complex multicomponent enzyme systems, the polyketide synthases (PKS), classed as type I, type II and type III [27]. From these, modular type I PKS complexes offer attractive prospects for producing novel metabolites through genetic engineering and combinatorial biosynthesis [28].

Despite exploration of bacterial communities from the underground river in Yucatán and its cenotes [29–31], there is little information on their biotechnological potential. By 2020, only 18 of the > 7000 mapped cenotes [1] had been assessed for their cultivable microbial diversity [3], and microorganisms from only six had been assessed for their biotechnological potential [3]; most studies have focused on inland cenotes, whereas the present study concerns a coastal cenote, Pol-Ac, with the aim of characterizing 49 Gram-positive bacteria isolated from sediment samples. This work provides insights into the diversity, enzymatic activity, and antimicrobial potential of bacteria inhabiting these unexplored habitats.

## Materials and Methods

### Materials

Chemicals, product standards, and solvents were obtained at the highest purity available from Sigma-Aldrich (St. Louis, US).

### Sampling Site

Pol-Ac is an open coastal cenote within a mangrove environment in the El Palmar State Reserve in Yucatán, Mexico (21.0816118, -90.2029322), ~ 780 m east of the Gulf of Mexico (Fig. S1). It is cylindrical, with a cave opening of 4000 m^2^, and reaches a depth of 63 m at its lowest point. Water temperature, salinity, dissolved oxygen, and pH were determined continuously every 2 s to a depth of 53.5 m with a multiparameter EXO1 water probe (Xylem Analytics, Norway).

### Microorganism Isolation

Marine sediment samples were collected in April 2021 under three different depths; a low depth at 14 m which is exposed to high solar radiation, a medium depth at 24 m which is exposed to low solar radiation and a deep depth at 54 m which is exposed to no solar radiation. Three replicates were collected from each sampled point in sterile hermetic seabags. Samples were cooled immediately on ice and stored at 4 °C until further processing. Additionally, 40 L of water from the surface was collected, stored at 4 °C, and later sterilized in an autoclave to be used in preparing the media.

All subsequent steps were performed under sterile conditions. Sediment samples were resuspended 1:10 in sterilized water from Pol-Ac. Thereafter, serial dilutions up to 10^­‑7^ were prepared and streaked onto four different agar plate media. The first employed A1 media (10 g L^−1^ starch, 4 g L^−1^ yeast extract, 2 g L^−1^ peptone, 16 g L^−1^ agar) [32] with surface water from Pol-Ac (to ensure the exact composition of salts and nutrients), the second one A1 media with marine sea water from the coast of Sisal (A1_m_ agar plates), the third one Brain Heart Infusion agar (8 g L^−1^ Brain and Heart Infusion, 5 g L^−1^ peptic digest of animal tissue, 16 g L^−1^ pancreatic digest of casein, 5 g L^−1^ sodium chloride, 2 g L^−1^ glucose, 2.4 g L^−1^ disodium hydrogen phosphate and 13.5 g L^−1^ agar) prepared with ddH_2_O to target more generalist bacteria, and the fourth one only agar (16 g L^−1^) with surface water from Pol-Ac. All plates were incubated at 27 °C for 1 to 2 weeks. Single colonies, selected on the basis of their morphology, pigmentation and the formation of a halo around the colony [33], were subcultured on A1_m_ agar until axenic cultures were obtained.

Gram-positive strains, determined via the nonstaining KOH method [34], were inoculated in 50 mL of A1_m_ liquid medium and incubated for four days at 27 °C. These liquid cultures were homogenized, mixed 1:1 with 70% glycerol and stored as glycerol stocks at -80 °C until further use. In total, 49 strains were preserved in glycerol stocks.

### DNA Extraction and 16 S Sequencing

Genomic DNA extraction used the Quick-DNA Fungal/Bacterial Microprep Kit (Zymo Research, Irvine, US) and followed the manufacturer’s instructions. DNA concentrations were quantified with a Nanodrop One spectrophotometer (Thermo Scientific, Waltham, US), and DNA integrity was assessed on agarose gel (1%). Amplification of the 16 S rDNA gene used the universal primer pair 27 F (5′-AGAGTTTGATCCTGGCTCAG-3′) and 1492R (5′-TACGGYTACCTTGTTACGACTT-3′) [35]. The PCR was performed on a CFX96 Real-Time System thermal cycler (Bio-Rad, Hercules, US) using the following protocol: Initial denaturation for 5 min at 95 °C, followed by 30 cycles of 40 s at 95 °C for denaturation, 50 s at 60 °C for annealing, 40 s at 72 °C for extension, and 10 min at 72 °C for a final extension. The amplified products were visualized by electrophoresis on agarose gel (1%) using the Biorad Molecular Imager Gel Doc XR + System (Bio-Rad, Hercules, US) (Fig. S2). The amplicons were sequenced by the Sanger technique at the Institute of Biotechnology of the National Autonomous University of Mexico (UNAM, Mexico City).

### Phylogenetic Analysis

The partial 16 S DNA sequences obtained (503–970 bp) were trimmed with SnapGene v6.2.1 software (GSL Biotech, US) and were compared against the NCBI database via a BLAST search. Based on sequence identity the nearest neighbors and nearest type strains to those neighbors were selected to accurately classify the genera of the strains (Table S2). A phylogenetic tree was generated with the default mode of the online tool phylogeny.fr, which uses MUSCLE (v. 3.8.31) for multiple sequence alignment, Gblocks (v. 0.91b) for alignment refinement, PhyML (v. 3.1/3.0 aLRT) for phylogeny and finally TreeDyn (v. 198.3) for tree drawing [36].

### Determination of Thermotolerant Strains

The influence of increasing temperatures on bacterial growth was assessed on strains growing on agar plates. Therefore, test tubes with liquid A1_m_ media were inoculated with glycerol stocks of the strains and grown at 27 °C for five days. 2 µL of these cultures were drop spotted onto one square agar plate containing A1_m_ agar (Fig. S3) in biological duplicates. After incubation for 5 days at 25 °C, 35 °C, 45 °C, 55–65 °C, the plates were examined for bacterial growth.

### Marine Water Requirement for Growth

The reliance of the bacterial strains on marine water for growth was investigated in an agar plate assay. Test tubes with liquid A1_m_ media were inoculated with glycerol stocks of the strains and grown at 27 °C for five days. 2 µL of these cultures were drop spotted in biological duplicates onto one square agar plate containing either A1_m_ agar or A1 agar prepared with bidistilled water, ddH_2_O (A1_ddH2O_ agar plates). After incubation for 5 days at 27 °C, the plates were examined for bacterial growth.

### Agar Plate Screening of Extracellular Hydrolytic Enzymes

To evaluate extracellular enzymatic activity of the isolated strains, agar plate assays were conducted with the respective substrates, employing drop spots. The composition of each culture medium for the assays is described below. In contrast to the commonly used formulation with ddH_2_O, all agar plates were prepared with filtered marine water to support the halophilic nature of the bacterial strains. Media were autoclaved for 20 min at 121 °C and plates stored at 4 °C until further use. Test tubes with liquid A1_m_ media were inoculated with glycerol stocks of the strains and grown at 27 °C for five days. On each agar plate, six different strains were spotted by adding 5 µL of the corresponding culture and incubating at 27 °C for five days, unless stated otherwise. All assays were performed in biological duplicates. The hydrolytic activities of the corresponding strains were expressed as level of enzymatic activity (LEA), as described previously [37, 38], by dividing the diameter of the clearance zone by the diameter of the colony in millimeters. For all agar plate assays, *Escherichia coli* XL1-blue was the negative control since *E. coli* is not only a poor secretor of enzymes [39], but also tests negative for extracellular amylase [40], cellulase [41], chitinase [42], lipase [43], gelatinase [44] and protease [45].

### Amylase Activity

A1_m_ agar plates were used to determine the level of amylase activity. After incubation for five days, the plates were flooded with Lugol’s iodine solution (Materiales y Abastos Especializados S.A. de C.V., Mexico City, MX) for 1 min. A clear halo around a colony would indicate starch hydrolysis, thereby confirming amylase activity [37].

### Cellulase Activity

Cellulase activity was determined on carboxymethylcellulose (CMC)-based agar plates [38] containing CMC (5 g L^−1^), NaNO_3_ (2 g L^−1^), K_2_HPO_4_ (1 g L^−1^), MgSO_4_ (0.5 g L^−1^), KCl (0.5 g L^−1^), peptone (0.2 g L^−1^) and agar (18 g L^−1^). After autoclaving, CMC precipitates partially in marine water. Thus, the CMC degradation can be monitored without the need of a staining solution. After incubation for five days, the plates were examined. A clear halo around a colony would indicate CMC hydrolysis, thereby confirming cellulase activity.

### Chitinase Activity

Chitinase activity was determined on chitin agar plates [46] with a simplified formula: colloidal chitin (100 g L^−1^, equal to 5 g dried chitin L^−1^), yeast extract (0.4 g L^−1^), peptone (0.2 g L^−1^) and agar (16 g L^−1^). Colloidal chitin was prepared according to Hsu and colleagues [47]. The degree of chemical modification of chitin is higher than that of cellulose and starch, so a ten-day incubation period was chosen for examination. A clear halo around a colony would indicate chitin hydrolysis, thereby confirming chitinase activity.

### Lipase Activity

Olive oil agar plates [48] were used to determine lipase activity. Plates were prepared with olive oil (10 mL L^−1^), CaCl_2_ (1 g L^−1^), phenol red (0.1 g L^−1^) and agar (20 g L^−1^) and the pH was adjusted with NaOH to 7.3–7.4. Following a five-day incubation period, the plates were examined. The principle of the lipase agar plate assay hinges on a pH decrease from 7.3, representing the endpoint of the phenol red dye, to a slightly more acidic pH, inducing a distinctive color transition from red to yellow. This decrease in pH, attributed to the release of fatty acids formed by the lipase-catalyzed breakdown of olive oil, serves as the indicator of lipase activity. Thus, a discernible yellow halo encircling a colony would indicate olive oil hydrolysis, thereby confirming lipase activity.

### Gelatinase Activity

Gelatinase activity was assessed on gelatin agar plates [44]: gelatin (12 g L^−1^), yeast extract (1 g L^−1^), peptone (4 g L^−1^), and agar (18 g L^−1^). After incubation for five days, the plates were treated with a saturated (NH_4_)_2_SO_4_ solution, causing the precipitation of unhydrolyzed gelatin and thereby obscuring the corresponding agar plate areas. Subsequently, the plates were checked after five minutes, with a clear halo around a colony indicating gelatin hydrolysis, thereby confirming gelatinase activity.

### Protease Activity

Protease activity was determined on skimmed milk agar plates as prepared by Menasria and collaborators (Menasria et al. (2018): skimmed milk (10 g L^−1^), yeast extract (1 g L^−1^) and agar (20 g L^−1^). After incubation for five days, the plates were examined. A clear halo around a colony would indicate protein hydrolysis, confirming protease activity.

### Determination of Polyketide Synthases

To show the presence of polyketide synthase genes encoding for type I PKS enzymes, genomic DNA from each of the 49 cultivated strains was amplified via PCR using the two degenerate oligonucleotide primer pairs 5LL/4UU and KPF/KPR: 5LL (5’-GGRTCNCCIARYTGIGTICCIGTICCRTGIGC-3’) with 4UU (5’-MGIGARGCIYTICARATGGAYCCICARCARMG-3’) [49]; and DKF (5’-GTGCCGGTNCCRTGNGYYTC-3’) with DKR (5’-GCGATGGAYCCNCARCARYG-3’) [50]. Following amplification, presence of products was determined by visualizing in a 1% agarose gel. The anticipated amplicon products for both primer pairs were expected to be approximately 700 base pairs in length.

### Antimicrobial Activity Assessment

Extracts of all 49 strains were examined to assess their antimicrobial activity against the Gram-negative bacterium *E. coli* ATCC 35,218 and the Gram-positive bacterium *Staphylococcus aureus* (*S. aureus*) ATCC 6538. Antibiotic activities of all extracts and reference antibiotics (kanamycin and gentamycin) were tested with the broth dilution method based on the Clinical and Laboratory Standards Institute (CLSI) recommendations [51].

For the preparation of the extracts, the strains were reactivated from cryopreserved glycerol stocks on A1_m_ agar plates and a single colony was transferred to a 250 mL Erlenmeyer flask containing 50 mL of A1_m_ broth. Cultures were grown for 7 days, harvested and centrifuged at 4000 rpm. The supernatants were recovered, filtered through syringe filters of 0.8 μm, and lyophilized to generate dry powders, which were stored at 4ºC until further processing. Before performing the antimicrobial evaluation, 100 mg of each lyophilized powder were solubilized in ultrapur water by vigorous vortexing and then desalted by addition of ethanol, precipitating the solid salt. To remove salts, salted-out samples were centrifuged at maximum speed for 2 min. The supernatants were recovered in 2 mL Eppendorf tubes and dried under vacuo (Eppendorf® Concentrator Plus™ for microtubes). The obtained desalted samples were then employed to evaluate the antimicrobial activity. To perform the antibiotic experiments, 10 mg of each desalted sample were resuspended in 1 mL of 70% ethanol for use as a stock solution.

The activity assay used Mueller–Hinton broth medium with incubation at 150 rpm and 37ºC for the duration of the experiment. To perform the test, 5 µL of stock solution of an extract were added to 195 µL of pathogenic bacterial culture (final concentration 250 µg mL^−1^) at the beginning of the exponential growth phase (10^8^ CFU mL^−1^). Reference antibiotics were solubilized in water and added at a final concentration of 25 µg mL^−1^. Optical density at 600 nm was determined at the initial and final times of the experiment. The growth inhibition (%) of the bacterial pathogen strains was assessed by use of negative controls grown with the addition of 5 µL ethanol (70%) instead of bacterial extract stock solution. All the experiments were performed in triplicate and at least three independent experiments were recorded.

## Results

### Physicochemical Characteristics of Water in Pol-Ac

**Fig. 1:**
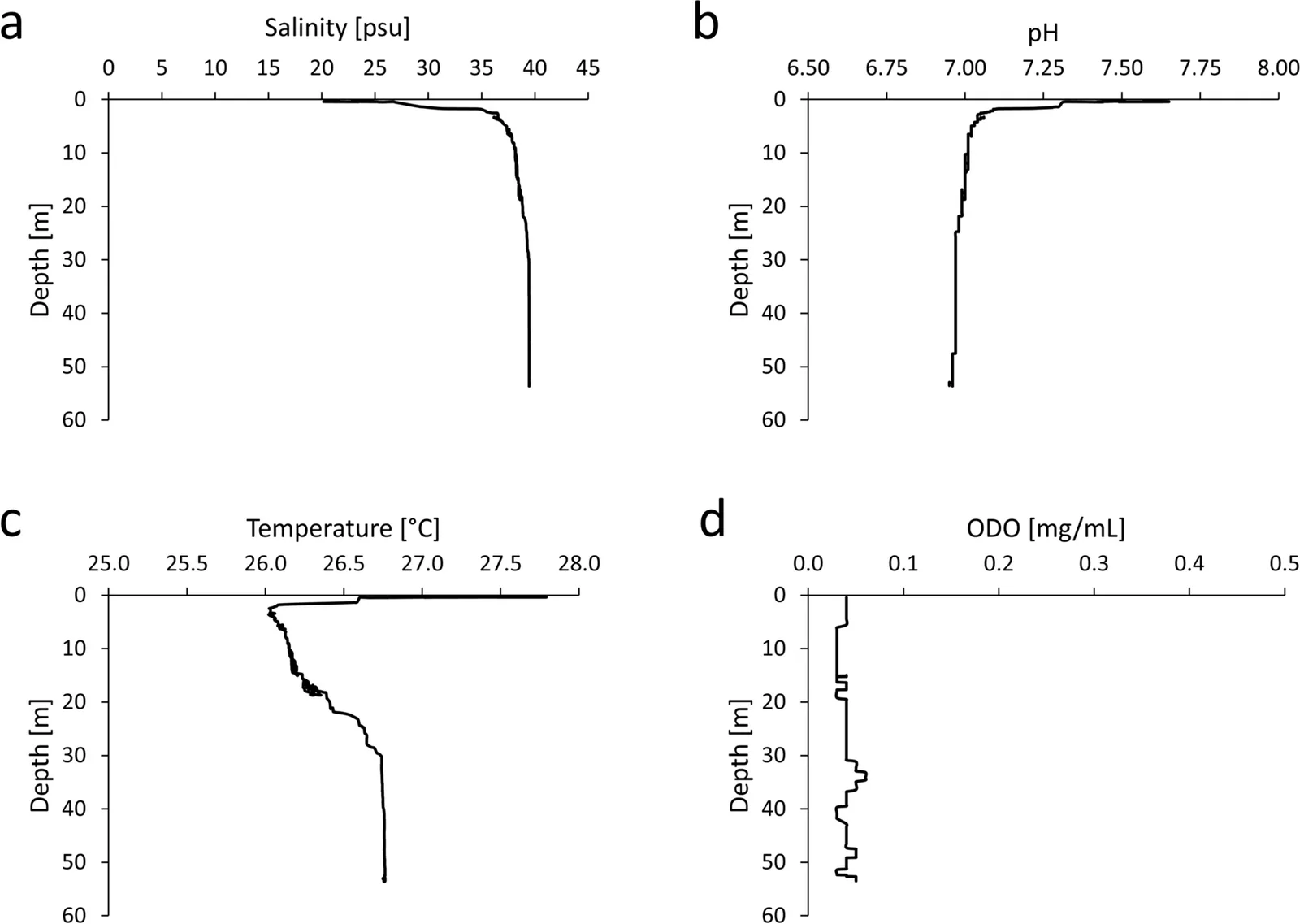
Physicochemical characteristics of water at different depths in Pol-Ac. **a** Salinity; **b** pH; **c** Temperature; **d** Dissolved oxygen (optical sensor)

Salinity at the surface of the water column in Pol-Ac was 20.9 psu (Fig. 1a). As depth increased, salinity rose rapidly to 36.0 psu at a depth of 2.5 m, and then continued to increase gradually to 39.5 psu at a depth of 53.5 m. Thus, Pol-Ac can be considered a thalassic environment. pH values decreased with depth (Fig. 1b): 7.59 at the surface and 7.1 within the saltwater layer at 2.5 m. Beyond this depth, a further decrease gradually reached 6.95 at a depth of 53.5 m.

The water temperature is 27.4 °C at the surface, decreases to 26.0 °C at 2.5 m, and gradually increases to 26.8 °C at a depth of 30.0 m, after which it remains constant (Fig. 1c). Dissolved oxygen concentrations ranged from a minimum of 0.03 mg L^−1^ to a maximum of 0.06 mg L^−1^ with a mean value of 0.04 ± 0.01 mg L^−1^, indicative of a hypoxic environment (Fig. 1d).

### Microbial Diversity of Pol-Ac

**Fig. 2:**
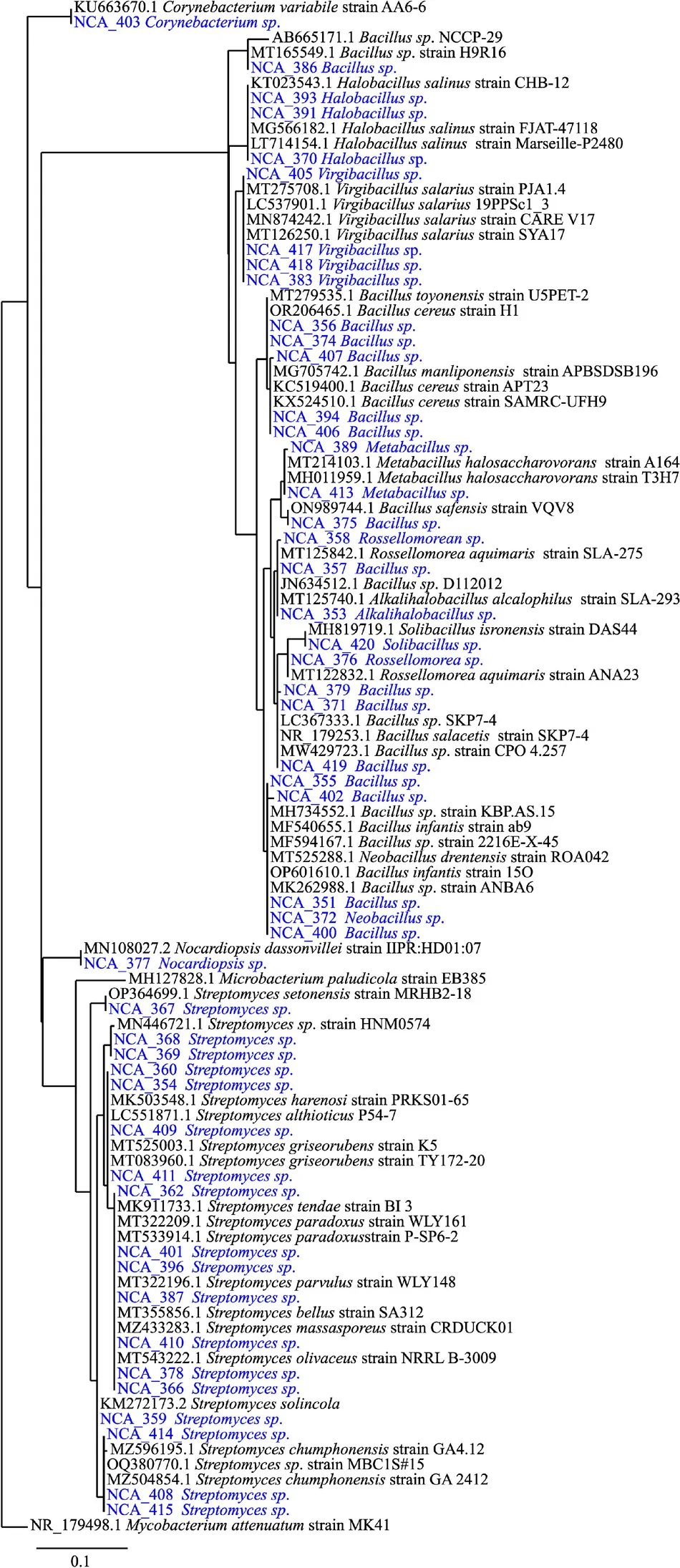
Phylogenetic tree of Gram-positive bacteria isolated from Pol-Ac (blue) with nearest BLAST neighbors (black) and an outgroup (*Mycobacterium attenuatum* strain MK41)

In total, 49 Gram-positive strains were isolated from the sediment of the cenote, whereby 44 strains were isolated from sediment collected at a depth of 14 m, four at a depth of 24 m and one from 54 m. Due to the limited number of strains isolated at medium and deep depths, a significant correlation between the depth of sample collection and strain affiliation, as well as enzymatic or antimicrobial activity was not possible. Further, of the 49 isolated strains, 39 were isolated from A1_m_ agar plates and 10 from A1 agar plates prepared with surface water from Pol-Ac. No Gram-positive bacteria could be isolated from either Brain Heart Infusion agar or plates containing only agar and surface water of Pol-Ac. The BLAST search showed identities between the partial gene sequences and the nearest neighbor that ranged from 98.1 to 100%. Of the 49 strains, 29 represented seven genera of the Bacillota: *Bacillus* (17 strains), *Virgibacillus* (4), *Halobacillus* (3), *Metabacillus* (2), *Solibacillus* (1) *Neobacillus* (1) and *Rossellomorea* (1). The other 20 strains represented three genera of the Actinomycetota: *Streptomyces* (18 strains), *Nocardiopsis* (1) and *Corynebacterium* (1) (Fig. 2, Fig. S4 and Fig. S5). All sequences were deposited in the NCBI GenBank database with the accession numbers OR844320-OR844368 (Table S2).

### Determination of thermo- and Halotolerant Strains

At the lower incubation temperatures (25 °C and 35 °C) all 49 strains grew robustly, in accordance with the measured water temperature range in Pol-Ac (26.0–27.4 °C); at 45 °C, the lower threshold for thermotolerance [52], 45 (91.8%) had substantial growth; at 55 °C, growth of 21 strains (42.9%) was characterized by reduced colony diameters ranging from 0.3 mm to 2 mm; and at 65 °C none grew. Therefore 21 of the strains isolated from Pol-Ac can be considered as moderately thermotolerant and 28 as mesophilic. Of the 21 moderately thermotolerant strains, 18 belonged to the Bacillota and 3 to the Actinomycetota.

All strains grew on the A1_m_ agar plates, with a salt concentration of 3.5%, classifying them as slightly halotolerant [53]. Of the 49 strains, 24 were not capable of growing on A1_ddH2O_ agar plates, indicating that these have a specific requirement for marine water and are likely halophiles [19]; 19 belonged to the Bacillota (six genera) and 5 to the Actinomycetota (to one genus).

#### Extracellular Hydrolytic Enzymes

**Fig. 3:**
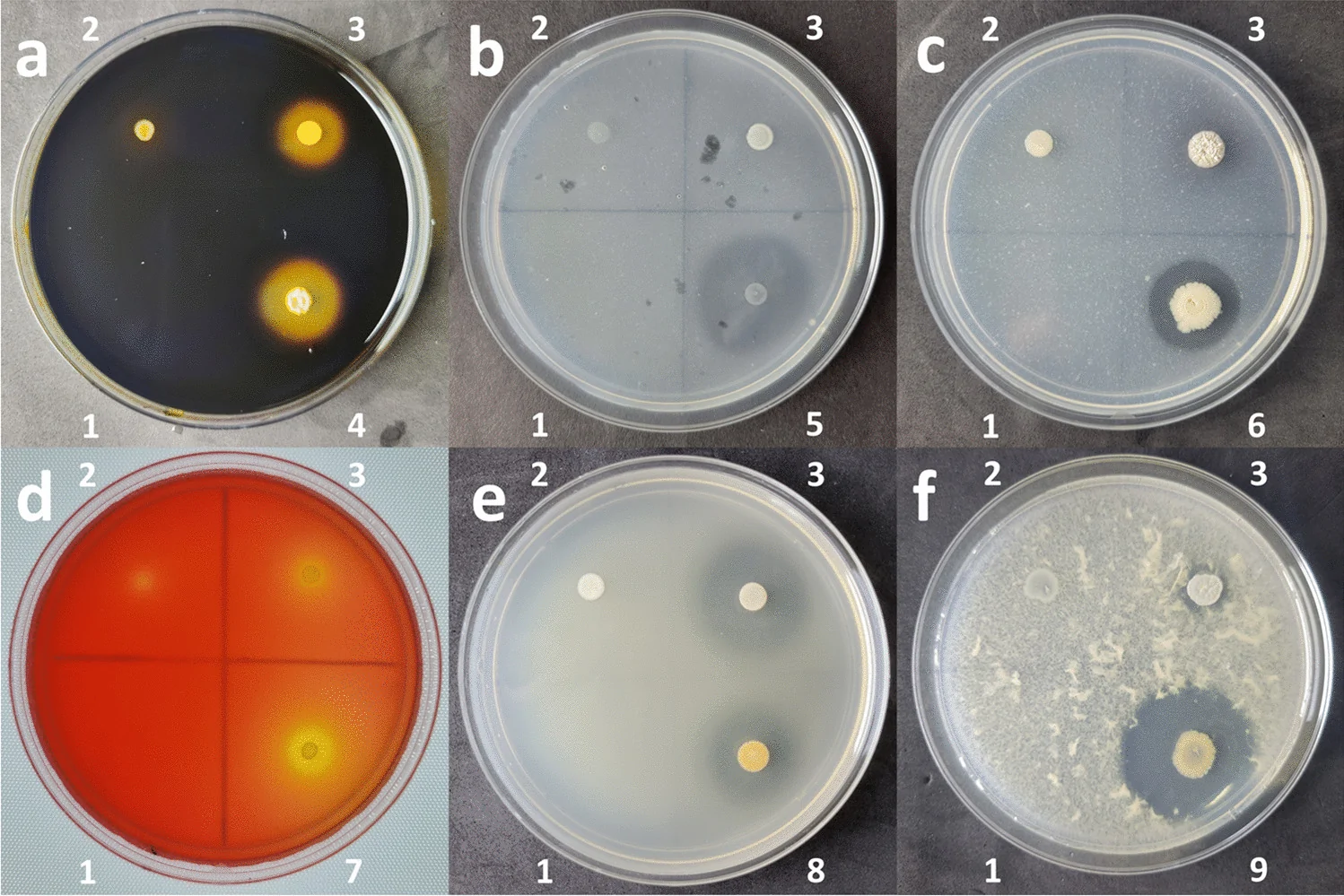
Representative agar plate assays for the detection of extracellular hydrolytic enzymes. All plates used an uninoculated empty control (1), *E. coli* XL1-blue as a negative control (2), and *Streptomyces* sp. NCA*_*378 (3), which exhibited activity for all six assays. Colonies 4–9 represent strains with very high activities. **a** Amylase assay with *Solibacillus* sp. NCA_420 (4); **b** Cellulase assay with *Halobacillus* sp. NCA_370 (5); **c** Chitinase assay with *Bacillus* sp. NCA_375 (6); **d** Lipase assay with *Bacillus* sp. NCA_353 (7); **e** Gelatinase assay with *Bacillus* sp. NCA_358 (8); **f** Protease assay with *Bacillus* sp. NCA_419 (9)

**Fig. 4:**
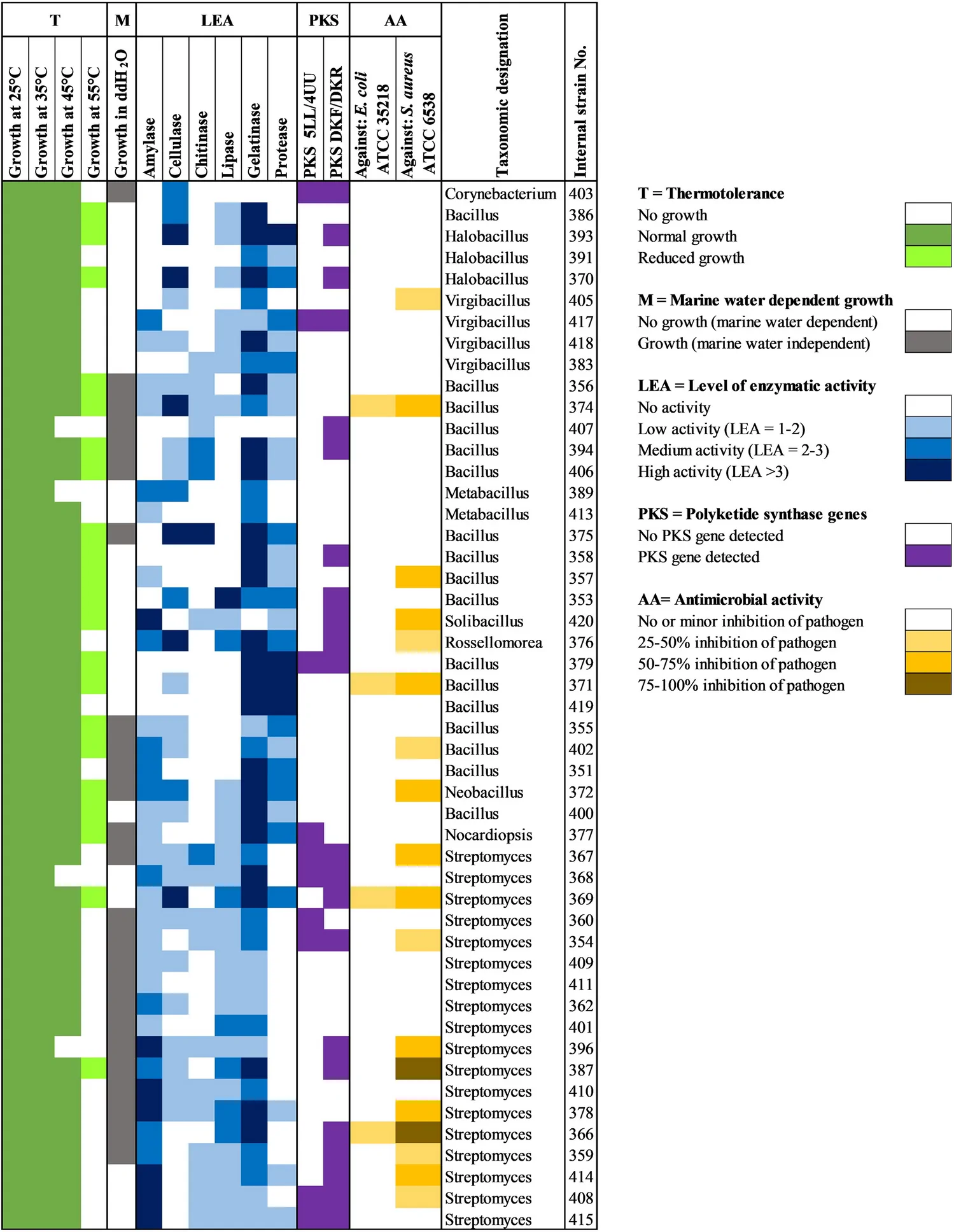
Activity profile of the 49 bacteria isolated from the cenote Pol-Ac. From left to right: **T**: thermotolerance (light green to dark green); **M**: Marine water dependent for growth (grey), **LEA**: level of enzyme activity (light blue to dark blue); **PKS**: assessment of type I PKS genes (purple); **AA**: antimicrobial activity (light yellow to brown); taxonomic designation and internal strain number (NCA)

Screening of bacterial isolates for extracellular hydrolytic enzymes (Fig. 3) showed that each of the 49 strains could produce at least one of these enzymes (Fig. 4); 47 could produce at least two extracellular enzymes; 41 at least three; 33 at least four; 15 at least five; and 2 could produce all six extracellular enzymes. Gelatinase activity was the most prevalent, seen in 46 of the isolates; amylase activity in 33; lipase activity in 31; cellulase and protease activity each in 29 of the strains; and chitinase activity in only 19.

Since marine water was used for all six agar plate assays, all detected enzymes and their corresponding activities are halotolerant. Actinomycetota, on average, showed a higher cumulative enzyme activity rate (70.0%) than that observed in Bacillota (59.2%). In addition to being the most frequently isolated genus (*n* = 18), all *Streptomyces* strains exhibited enzymatic activity in the forms of amylase, lipase, and gelatinase; 11 of the *Streptomyces* strains showed chitinase activity; 10 showed cellulase activity; and 4 showed protease activity. From Pol-Ac, *Streptomyces* NCA_378 showed activities of all six extracellular enzymes: high for amylase and gelatinase; medium for lipase; and low for cellulase, chitinase and protease. Hence, this strain merits further study.

In contrast, *Bacillus*, the second most commonly isolated genus (*n* = 17), displayed a more varied distribution of enzymatic capabilities: 16 of the *Bacillus* strains exhibited gelatinase activity; 15 showed protease activity; 11 showed cellulose; 7 amylase; 6 chitinase; and 4 lipase. From Pol-Ac, *Bacillus* NCA_374 displayed high biotechnological potential across all six extracellular enzymes, with high cellulase activity, medium gelatinase activity, and low activity levels for amylase, chitinase, lipase, and protease. In addition, strain *Halobacillus* NCA_393 showed high activity of cellulase, gelatinase, and protease activities, and *Bacillus* NCA_375 showed high activity in cellulase, chitinase, and gelatinase. Hence, these two Bacillus strains, too, merit further biotechnological study.

### Detection of Type I PKS Genes

In the current study, putative type I polyketide synthase (PKS) genes were identified in 24 of the isolated strains; 22 of these showed a PCR product with the KPF/KPR primer pair, and 10 with the 5LL/4UU primer pair. Of the 20 Actinomycetota strains, 14 (70.0%) showed a PCR product with the PKS primers, whereas of the 29 Bacillota strains only 10 (34.5%) showed a product.

#### Determination of Antimicrobial Activity

Four strains (8.2%) slightly inhibited the growth of *E. coli* ATCC 35,218 (25–50% inhibition in comparison to negative control) (Fig. 4). All four strains that slightly inhibited *E. coli* ATCC 35,218 were also active against *S. aureus* ATCC 6538. In contrast to the low number of strains inhibiting *E. coli*, 18 (36.7%) inhibited growth of *S. aureus* ATCC 6538, 10 (20.4%) of these showing medium inhibition (50–75%). Two strains, *Streptomyces* NCA_366 and *Streptomyces* NCA_387, achieved high levels of inhibition (75–100%; Fig. 4) and merit further study. Higher percentages of Actinomycetota (50%) than Bacillota (27.6%) showed inhibition against one of the two tested pathogens. These findings are promising and suggest that bacteria isolated from cenotes such as Pol-Ac have the potential to produce antimicrobial compounds with activity against Gram-positive bacteria such as *S. aureus* and should therefore be further investigated for secondary metabolites.

## Discussion

This study focused on the bioprospection of cultivable Gram-positive bacteria isolated from sediment samples of the cenote Pol-Ac. In contrast to other coastal cenotes, Pol-Ac lacks a freshwater layer on its surface due to a surface salinity of 20.9 psu and a halocline at a shallow depth of only 0.4 m. For instance, the coastal cenotes Tábano [54] and Na’ach Wennen Ha [55], both within 1 km of the Caribbean Sea, have surface salinities of 2 psu and 0.5 psu, and much deeper haloclines at 11 m and 5 m, respectively. With an average water temperature of 26.4 °C, Pol-Ac follows the trend of other coastal cenotes like Tabano and Odyssey [54] during this time of the year [5, 56].

However, the dissolved oxygen concentrations along the whole water column were remarkably low, with a maximum of only 0.06 mg L^−1^. A study involving 30 coastal cenotes of the Yucatán Peninsula reported minimum oxygen concentrations to be more than 10 times higher than Pol-Acs [5]. Thus, the low oxygen levels correlated with the high salinity levels and with the eutrophication conditions [57]. The scarcity of macroscopic organisms in this cenote can be attributed to these low oxygen concentrations, as has been shown for hypoxic costal ecosystems [58]. These parameters suggest that Pol-Ac may host a unique microbial community that has adapted to these extreme conditions.

Previous studies of bacterial diversity in cenotes have reported a majority of Gram-negative isolates (80–90%) [4, 29]. However, among Gram-positive bacteria, the two most abundant phyla found by Escobar-Zepeda [29] were Bacillota and Actinomycetota, in the water and plant roots of a coastal cenote, similar than in our findings from Pol-Ac sediments. Indeed, Bacillota and Actinomycetota, found ubiquitously in soil and water ecosystems, actively contribute to the maintenance of ecological balance by efficiently degrading both organic and inorganic matter in their surroundings [59–61]. Further, the presence of *Virgibacillus* and *Halobacillus*, frequently found in saline environments [62, 63], is consistent with the marine-like nature of the sampling site.

The unexplored microbial biodiversity found in cenotes offers the potential for the discovery of novel enzymes that could have various applications in industries. Thus, one of the aims of this study was the exploration of extracellular hydrolytic enzymes secreted by the 49 isolated Gram-positive bacteria. Indeed, all the isolated strains were capable to produce at least one out of six of the screened enzymes. The present findings align with previous studies that reported the production of extracellular hydrolytic enzymes, including amylase, cellulase, chitinase, protease, gelatinase, and lipase, by marine coastal *Streptomyces* [38, 64] and marine *Bacillus* strains [65–67], which collectively accounted for 71.4% of the isolated strains in this study.

Another aim of the current work was the detection of PKS I genes of the isolated strains as well as the determination of their antimicrobial activity against the two model pathogen strains *E. coli* ATCC 35,218 and *S. aureus* ATCC 6538. PKS I amplicons were more abundant in the Actinomycetota group (70.0%) in comparison to the Bacillota one (34.5%). These results agree with reports of Actinomycetota, particularly *Streptomyces* species, as important producers of secondary metabolites, including pharmaceutically relevant polyketides [26, 68]. Nevertheless, PKS genes and their associated metabolites have also been found in Bacillota, such as species of *Bacillus* [69]. Further, 8.2% of the strains inhibited the growth of the pathogen *E. coli* ATCC 35,218 and 36.7% inhibited the growth of the Gram-positive pathogen *S. aureus* ATCC 6538. Low numbers of strains with activity against *E. coli* are expected, given the inherent resistance of Gram-negative bacteria to many antibiotics due to the presence of an outer membrane that restricts the penetration of many molecules into the cell [70]. Considering the remarkable capacity of Actinomycetota, especially Streptomyces, to produce abundant antimicrobial compounds, it is unsurprising to observe a higher percentage of antimicrobial activity in Actinomycetota (50.0%) compared to Bacillota (27.6%) [71–74]. Of the 49 strains, 24 showed a PCR product with the PKS I primers used for amplification, and 18 strains showed antimicrobial activity. However, only 11 strains showed both PKS I gene product amplification and antimicrobial activity. There was no significant correlation (chi-square 1.676; *p* = 0.196) between type I PKS gene product amplification and antimicrobial activity. This suggests that factors other than type I PKS genes, such as type II or type III PKS, non-ribosomal peptide synthetase genes [75] or other secondary-metabolite biosynthetic pathways, may contribute to the observed inhibition of these pathogens. Moreover, strains displaying amplicons for type I PKS genes, yet lacking antimicrobial activity against the two tested pathogens, may potentially yield polyketides that are active against pathogens not examined in the course of this study.

The present study emphasizes the potential of the microbial communities in cenote sediments of the Yucatán Peninsula to be a novel source of biotechnologically important bacteria. It demonstrates that the characteristics of the coastal cenote Pol-Ac form an environment that is ideal for further studies regarding the cultivation of halotolerant bacteria with hydrolytic enzymes of industrial value and potent antimicrobial compounds against known bacterial pathogens. Additionally, this study is the first report on the bioprospection of Gram-positive bacteria isolated from sediments in a cenote of the Yucatán Peninsula.

## Electronic Supplementary Material

Supplementary Material 1
