# Supplementary material for: Diversity and Bioprospection of Gram-positive Bacteria Derived from a Mayan Sinkhole
Source: Microb Ecol. 2024 May 29;87(1):77. doi: 10.1007/s00248-024-02392-1 (PMC11133088; doi:10.1007/s00248-024-02392-1)
Supplement: Supplementary file 1 — Supplementary Material 1 [file 248_2024_2392_MOESM1_ESM.pdf]

Microbial Ecology

Diversity and bioprospection of Gram-positive bacteria derived from a Mayan sinkhole

Julian L. Wissner[a], José Carlos Parada-Fabián[a], Norma Angélica Márquez-Velázquez[a], Wendy Escobedo-Hinojosa[a], Susana P. Gaudêncio[b,c], Alejandra Prieto-Davó[a,*]

[a]Unidad de Química en Sisal, Facultad de Química, Universidad Nacional Autónoma de México, Puerto de abrigo s/n, 97356 Sisal, Yucatán, México

[b]Associate Laboratory i4HB, Institute for Health and Bioeconomy, NOVA Faculty of Sciences and Technology, NOVA University of Lisbon, 2819-516 Lisbon, Portugal

[c]UCIBIO, Applied Molecular Biosciences Unit, Chemistry and Life Sciences Departments, NOVA Faculty of Sciences and Technology, NOVA University of Lisbon, 2819-516 Lisbon, Portugal

*Corresponding author

E-Mail address: apdavo@unam.mx

Tel.: +52 55 5622 6710 ext. 7110

Table of Contents

1. Satellite image of Pol-Ac....3
2. Amplified PCR product of the 16S rRNA region.... 4
3. Evaluation of thermotolerant bacteria.... 7
4. Taxonomic designation of the isolated strains.... 8

1. Satellite image of Pol-Ac

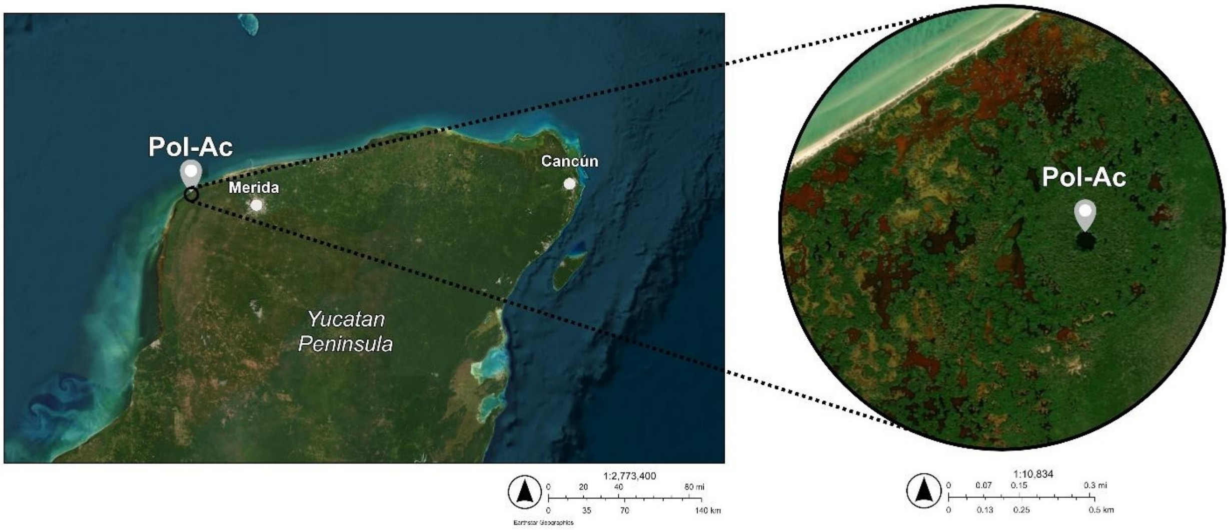

Fig. S1 Geographical location of the sampling site cenote Pol-Ac of the Yucatan Peninsula, Mexico. Maps were created with ArcGIS online software by ESRI (ESRI, 2022; Imagery Map of the Yucatan Peninsula. Satellite imagery from 18.03.2022. https://www.arcgis.com/index.html. Accessed 08.08.2023)

2. Amplified PCR product of the 16S rRNA region

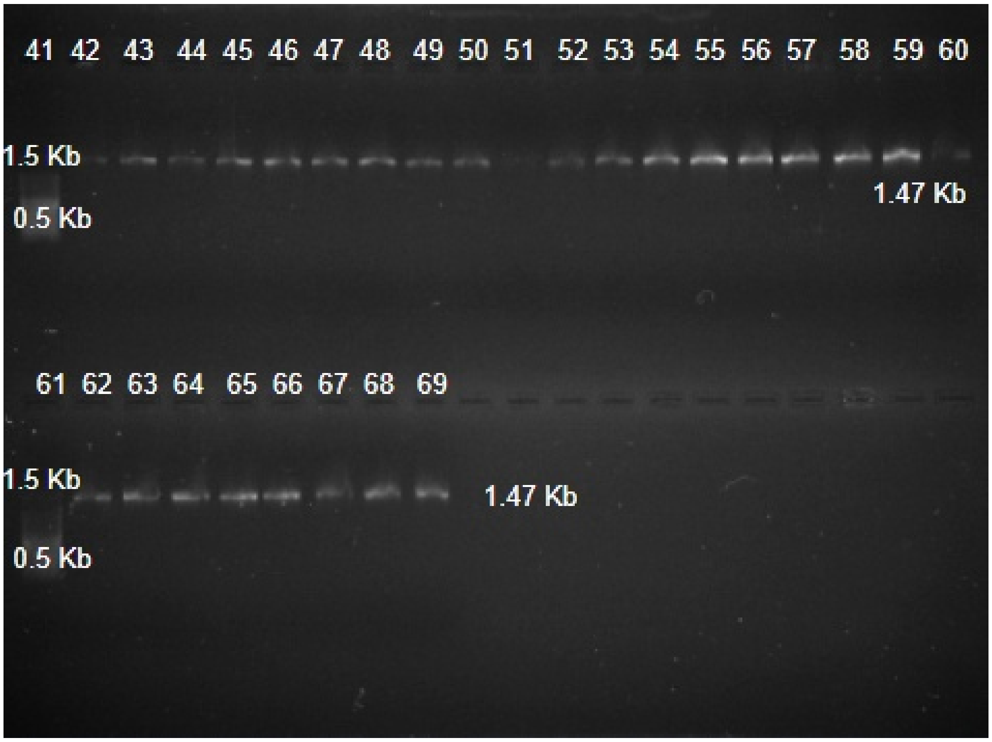

Fig. S2 Amplified PCR products (1.47 kb) of the 16S rRNA of each strain visualized on 1% agarose gel

Table S1 Number on agarose gel and the corresponding internal strain number NCA.

Agarose gel number	Internal strain number NCA
1	Marker
2	351
3	NA
4	353
5	354
6	355
7	356
8	357
9	358
10	359
11	360
12	NA
13	362
14	NA
15	NA
16	NA
17	366
18	367
19	368
20	369
21	Marker
22	370
23	371
24	372
25	NA
26	374
27	375
28	376
29	377
30	378
31	379
32	NA
33	NA
34	NA
35	383
36	NA
37	386
38	387
39	NA

40	389
41	Marker
42	NA
43	391
44	393
45	394
46	396
47	NA
48	400
49	401
50	402
51	403
52	NA
53	405
54	406
55	407
56	408
57	409
58	410
59	411
60	NA
61	Marker
62	413
63	414
64	415
65	NA
66	417
67	418
68	419
69	420

NA: Not Applicable; undetermined strain, not part of this study

3. Evaluation of thermotolerant bacteria

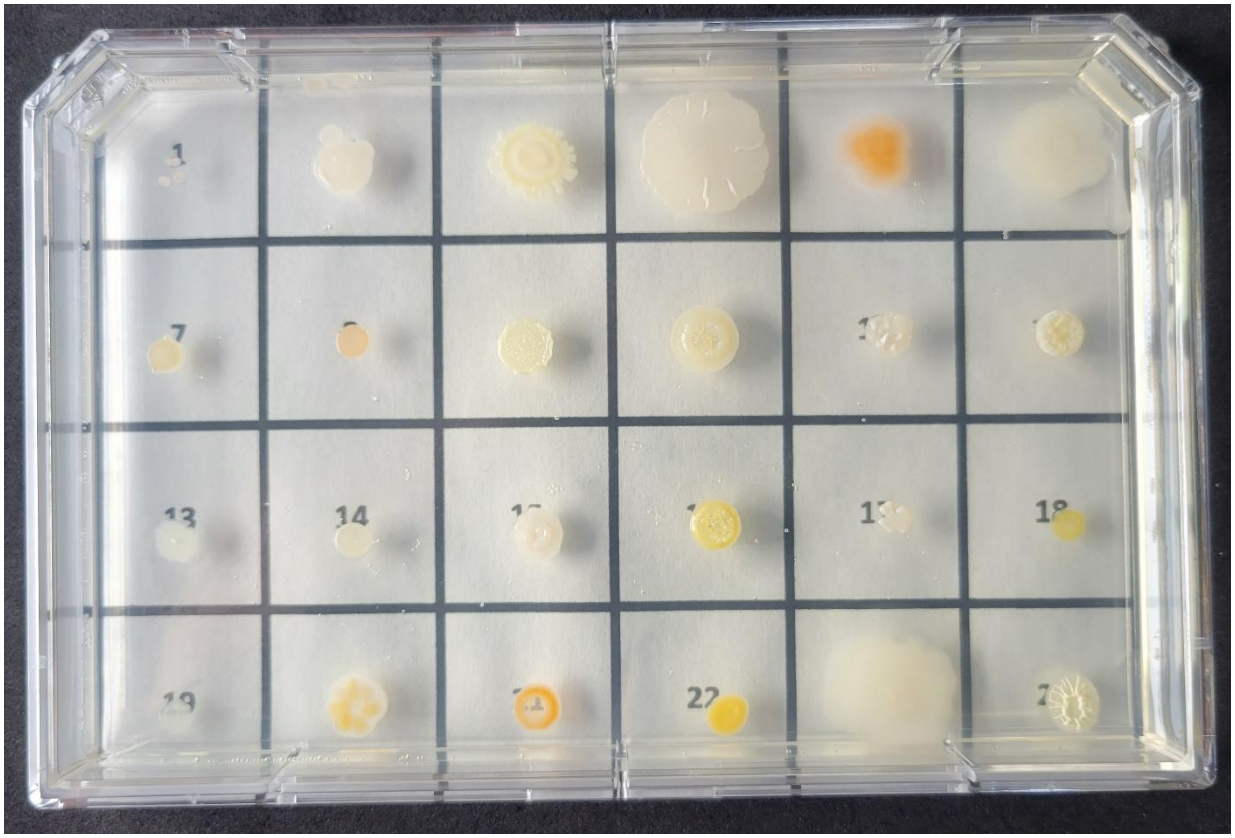

Fig. S3 Exemplary agar plate for the evaluation of thermostability, grown at 25° for five days

4. Taxonomic designation of the isolated strains

Table S2 Taxonomic designation of the 49 isolated bacteria with the NCBI access number and the corresponding identity in percent.

Strain No. NCA	GenBank accession No.	Taxonomic designation	GenBank accession No. of closest neighbor	Sequence identity [%]
351	OR844320	*Bacillus sp.*	MF594167.1	100
353	OR844321	*Bacillus sp.*	MN036336.1	100.0
354	OR844322	*Streptomyces sp.*	MK503548.1	99.4
355	OR844323	*Bacillus sp.*	MF540655.1	100.0
356	OR844324	*Bacillus sp.*	CP115307.1	100.0
357	OR844325	*Bacillus sp.*	JN634512.1	99.8
358	OR844326	*Bacillus sp.*	MK106062.1	99.3
359	OR844327	*Streptomyces sp.*	KM272173.2	100.0
360	OR844328	*Streptomyces sp.*	LC551871.1	98.8
362	OR844329	*Streptomyces sp.*	MZ433283.1	99.8
366	OR844330	*Streptomyces sp.*	MK911733.1	99.8
367	OR844331	*Streptomyces sp.*	OP364699.1	99.1
368	OR844332	*Streptomyces sp.*	MN446721.1	99.3
369	OR844333	*Streptomyces sp.*	LR217812.1	99.6
370	OR844334	*Halobacillus sp.*	MG566182.1	100.0
371	OR844335	*Bacillus sp.*	LC367333.1	99.9
372	OR844336	*Neobacillus sp.*	MT525288.1	100.0
374	OR844337	*Bacillus sp.*	MT279535.1	99.8
375	OR844338	*Bacillus sp.*	ON989744.1	99.5
376	OR844339	*Rossellomorea sp.*	MT122832.1	98.2
377	OR844340	*Nocardiopsis sp.*	MN108027.2	99.9
378	OR844341	*Streptomyces sp.*	MT533914.1	99.7
379	OR844342	*Bacillus sp.*	NR_179253.1	99.3
383	OR844343	*Virgibacillus sp.*	MN874242.1	99.8
386	OR844344	*Bacillus sp.*	AB665171.1	99.9
387	OR844345	*Streptomyces sp.*	MT355856.1	100.0
389	OR844346	*Metabacillus sp.*	MT214103.1	98.4
391	OR844347	*Halobacillus sp.*	LT714154.1	99.9
393	OR844348	*Halobacillus sp.*	KT023543.1	99.9
394	OR844349	*Bacillus sp.*	KC519400.1	100.0
396	OR844350	*Strepomyces sp.*	MT322196.1	100.0
400	OR844351	*Bacillus sp.*	MK262988.1	100.0
401	OR844352	*Streptomyces sp.*	MT322209.1	99.9
402	OR844353	*Bacillus sp.*	OP601610.1	99.3
403	OR844354	*Corynebacterium sp.*	KU663670.1	100.0
405	OR844355	*Virgibacillus sp.*	LC537901.1	100.0
406	OR844356	*Bacillus sp.*	KX524510.1	100.0
407	OR844357	*Bacillus sp.*	MG705742.1	98.1

408	OR844358	*Streptomyces sp.*	MZ504854.1	100
409	OR844359	*Streptomyces sp.*	MT525003.1	99.8
410	OR844360	*Streptomyces sp.*	MT543222.1	100
411	OR844361	*Streptomyces sp.*	MT083960.1	99.8
413	OR844362	*Metabacillus sp.*	MH011959.1	100
414	OR844363	*Streptomyces sp.*	OQ380770.1	99.7
415	OR844364	*Streptomyces sp.*	MZ596195.1	99.9
417	OR844365	*Virgibacillus sp.*	MT126250.1	99.8
418	OR844366	*Virgibacillus sp.*	MT275708.1	99.6
419	OR844367	*Bacillus sp.*	MW429723.1	100
420	OR844368	*Solibacillus sp.*	MH819719.1	98.9

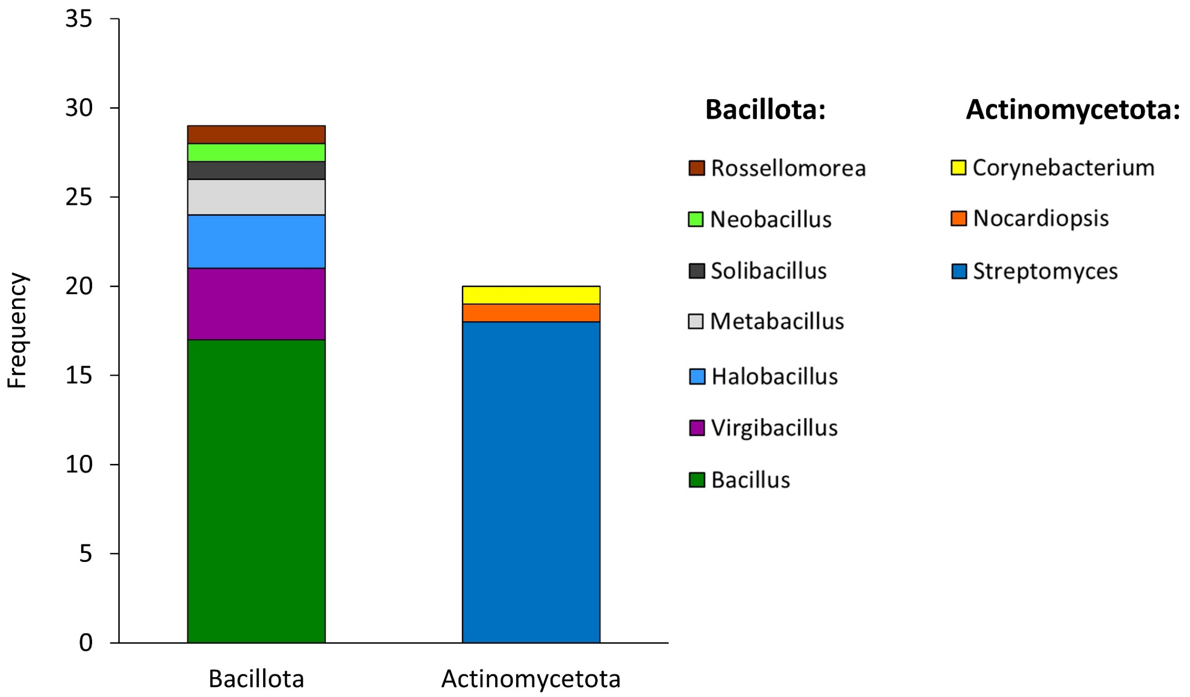

Fig. S4 Taxonomic designation of the sediment samples of Pol-Ac at the genus level

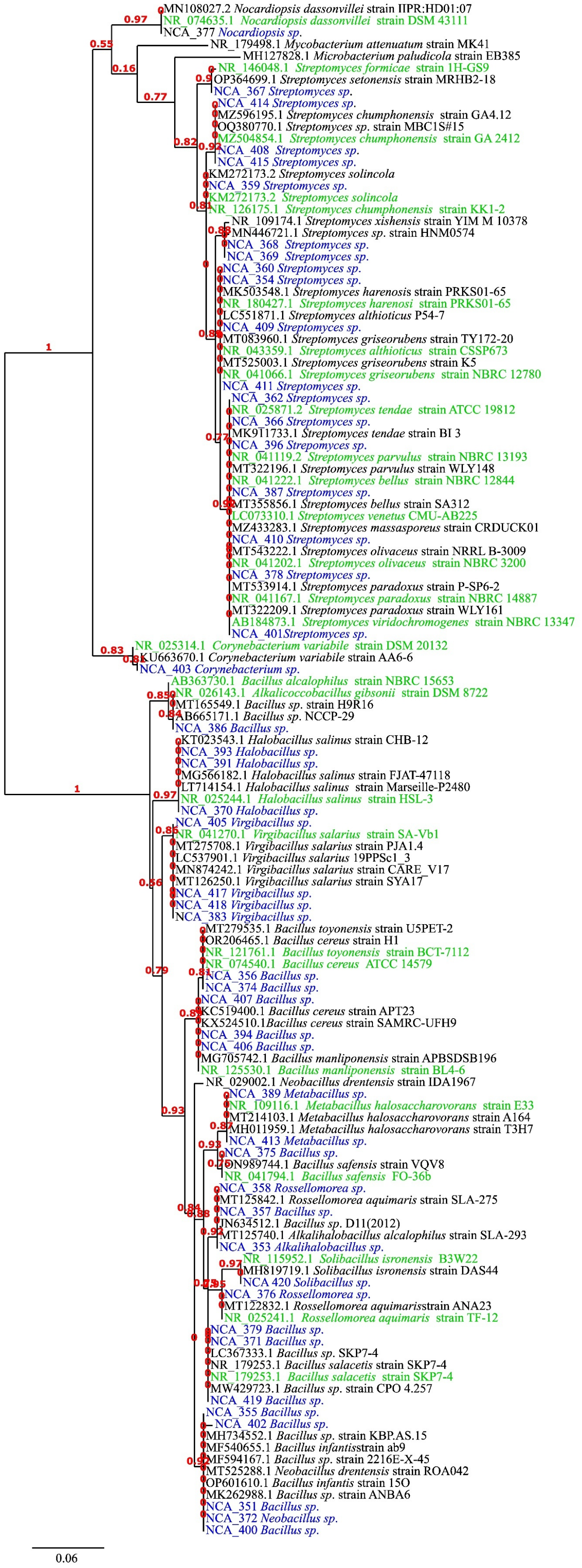

Fig. S5 Phylogenetic tree of Gram-positive bacteria isolated from Pol-Ac (blue) with nearest BLAST neighbors (black), their corresponding Type Strain (green), branch support values (red) and an outgroup (*Mycobacterium attenuatum* strain MK41)
